# Synchronization of ear-EEG and audio streams in a portable research hearing device

**DOI:** 10.3389/fnins.2022.904003

**Published:** 2022-09-01

**Authors:** Steffen Dasenbrock, Sarah Blum, Paul Maanen, Stefan Debener, Volker Hohmann, Hendrik Kayser

**Affiliations:** ^1^Auditory Signal Processing and Hearing Devices, Department of Medical Physics and Acoustics, University of Oldenburg, Oldenburg, Germany; ^2^Cluster of Excellence “Hearing4all”, University of Oldenburg, Oldenburg, Germany; ^3^Neuropsychology Lab, Department of Psychology, University of Oldenburg, Oldenburg, Germany

**Keywords:** hearing aids, mobile EEG, portable setup, timing, jitter, ear-EEG, cEEGrid, neuro-steered hearing device

## Abstract

Recent advancements in neuroscientific research and miniaturized ear-electroencephalography (EEG) technologies have led to the idea of employing brain signals as additional input to hearing aid algorithms. The information acquired through EEG could potentially be used to control the audio signal processing of the hearing aid or to monitor communication-related physiological factors. In previous work, we implemented a research platform to develop methods that utilize EEG in combination with a hearing device. The setup combines currently available mobile EEG hardware and the so-called Portable Hearing Laboratory (PHL), which can fully replicate a complete hearing aid. Audio and EEG data are synchronized using the Lab Streaming Layer (LSL) framework. In this study, we evaluated the setup in three scenarios focusing particularly on the alignment of audio and EEG data. In Scenario I, we measured the latency between software event markers and actual audio playback of the PHL. In Scenario II, we measured the latency between an analog input signal and the sampled data stream of the EEG system. In Scenario III, we measured the latency in the whole setup as it would be used in a real EEG experiment. The results of Scenario I showed a jitter (standard deviation of trial latencies) of below 0.1 ms. The jitter in Scenarios II and III was around 3 ms in both cases. The results suggest that the increased jitter compared to Scenario I can be attributed to the EEG system. Overall, the findings show that the measurement setup can time-accurately present acoustic stimuli while generating LSL data streams over multiple hours of playback. Further, the setup can capture the audio and EEG LSL streams with sufficient temporal accuracy to extract event-related potentials from EEG signals. We conclude that our setup is suitable for studying closed-loop EEG & audio applications for future hearing aids.

## 1. Introduction

Current neuroscientific discoveries have led to the concept of using brain signals as additional input to control audio signal processing in hearing devices (Slaney et al., 2020). In this process, correlates from the electroencephalography (EEG) recording are used to make statements about the user's mental state, such as auditory attention (O'sullivan et al., 2015) or listening effort (Bernarding et al., 2012; Haro et al., 2022). These can potentially be used to adjust the signal processing in the hearing device, for example, a beamformer that amplifies the attended speaker while suppressing the ignored ones (Aroudi and Doclo, 2019). This idea is commonly referred to as neuro-steered hearing devices, cognitively controlled hearing aids, or similar (O'Sullivan et al., 2017; Das et al., 2020; Geirnaert et al., 2021). Current developments in miniaturization and improving the wearability of (ear-centered) EEG suggest that the relevant neural signals can still be detected when using fewer electrodes and less spatial coverage compared to traditional cap EEG setups (Kidmose et al., 2012; Debener et al., 2015; Mikkelsen et al., 2015; Bleichner et al., 2016; Fiedler et al., 2017). This research is relevant to realize closed-loop applications on wearable devices with high wearing comfort.

To develop EEG-based hearing aid signal processing methods, a portable platform is required that offers the signal quality and timing accuracy required for closed-loop applications with audio and EEG signals. In Dasenbrock et al. (2021), we introduced a research platform, which combines the mobile Smarting EEG system and the so-called Portable Hearing Laboratory (PHL, Pavlovic et al., 2018). The PHL is a portable research hearing device that can fully replicate a hearing device. It uses the open-source open Master Hearing Aid (openMHA, Kayser et al., 2022) software for real-time, low-latency audio signal processing. The EEG data is received over a wireless network connection. The Lab Streaming Layer (LSL, Kothe et al., 2014) is used to synchronize audio and EEG data. LSL is a framework that can synchronize data streams from different devices by measuring their respective clock drift over a network connection to map the locally generated time stamps into one common timeline. First pilot timing and physiological tests showed that this setup's portable components could be used to extract event-related potentials (ERPs) using an Oddball paradigm (Dasenbrock et al., 2021).

The temporal synchronicity of audio and EEG data is crucial for such a setup. Alignment inaccuracies of audio and EEG data may lead to errors in the data analysis that may affect the predictive power of the following listening state analysis. Several sources of inaccuracy exist when aligning audio and EEG data. For example, there may be variations in the playback of the stimuli, i.e., differences between the software event markers and the actual playback of the device. Further, inaccuracies can occur when creating an LSL stream of continuous time-series data, such as the EEG. Generally, factors such as the operating system, drivers, and hardware performance will typically introduce variations in delays; thus, a latency variation (jitter) is always expected in real systems. Timing accuracy, especially stability (between sessions), must also be considered for online applications, which rely on a constant latency between EEG and audio.

Time synchronization has long been a challenge in the implementation of mobile EEG systems. When performing EEG experiments, the amount of jitter needs to be sufficiently small. The required temporal precision generally depends on the method used. It is particularly relevant for investigating time-averaged data. For instance, obtaining ERPs requires the extraction of EEG trials by epoching the data using event markers, which indicate a response-evoking feature of the auditory stream. Accurate time synchronization is crucial since it leads to an exact alignment when averaging over the single-trial responses, leading to high and sharp ERP components, i.e., specific peak amplitudes in the ERP (Williams et al., 2021). For wireless commercial mobile EEG systems, the problem of event-locking the EEG data has long been challenging, as they are often not designed for it. For instance, in early iterations of the wireless Emotiv EEG system, it was found that the built-in event-locking was unstable and did not produce high-quality ERPs (Hairston et al., 2014; Ries et al., 2014). Nowadays, mobile EEG hardware can also be used for ERP studies, as indicated by recent studies of the Emotiv EEG system (Williams et al., 2021). Further, fully mobile smartphone-based systems have already been successfully coupled with mobile Smarting EEG systems (mBrainTrain, Belgrade, Serbia), which could be used for extracting ERPs outside the laboratory (Debener et al., 2015; Blum et al., 2017; Hölle et al., 2022). Building upon the smartphone-based approach, the setup employed in this work focuses on hearing aid applications, leveraging the real-time capabilities of the PHL. The setup presented by our group in Dasenbrock et al. (2021) was extended by the possibility of sending single software event markers, which enabled us to perform a comprehensive timing analysis to evaluate its suitability for research into closed-loop hearing devices with EEG.

The timing precision of the setup was systematically evaluated in several timing test scenarios, that address the different components of the system. As the setup is composed of two completely independent components, namely the PHL and the Smarting EEG system, the timing precision of the setup is assumed to be composed of the timing accuracy of the PHL and the timing accuracy of the EEG system. The accuracy of these two systems was examined separately in timing test scenarios I and II. Timing test scenario I examines the PHL's ability to create a precise software marker that marks the playback time of an acoustic stimulus, i.e., the event marker. In this test, the event marker time is compared to the actual audio playback of the PHL. Timing test scenario II examines how precisely the EEG system samples and time stamps an incoming signal by comparing the sampled and time stamped signal from the EEG system against a known reference. A third test, i.e., timing test Scenario III, was conducted to measure the whole system's timing, as it would be present in a real EEG measurement. In this final test, a reference signal generated by the PHL is compared to the sampled and time stamped signal of the EEG system. To investigate differences in the timing accuracy between shorter and longer durations, all timing tests were performed for 15 min and 3 h. No human subjects were used in the tests, as their introduction would lead to additional between-subject variability (Intriligator and Polich, 1995), as well as measurement variability (Callaway and Halliday, 1973). Thus, this study was kept purely technical, focusing on the effects of the measurement devices.

## 2. Materials and methods

In the following two sections, the setup and its technical features as well as the timing tests are described in detail.

### 2.1. Setup

**Figure 1:**
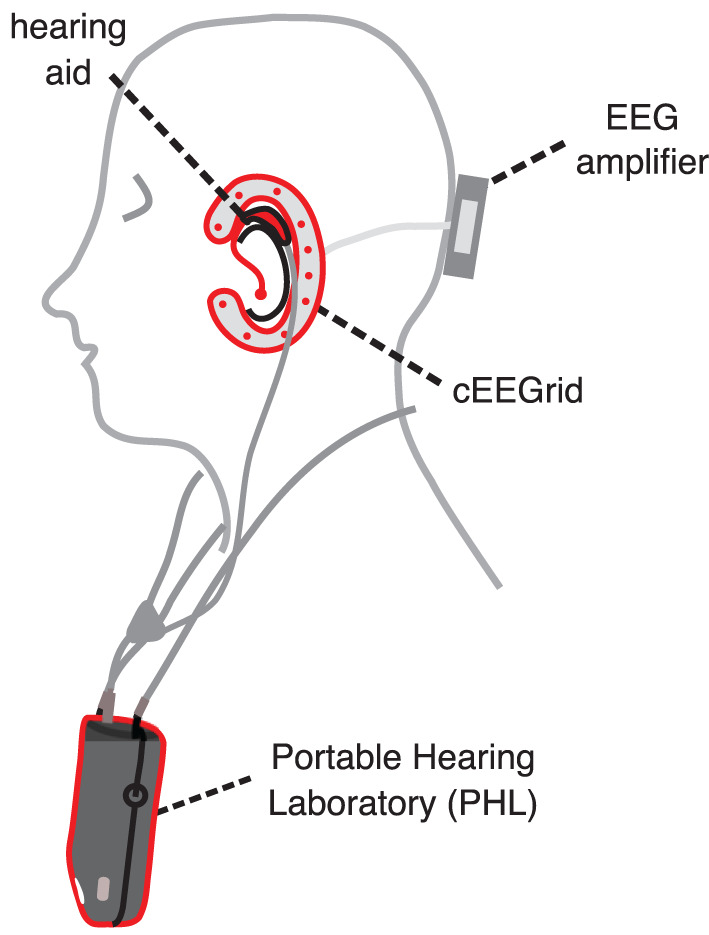
Sketch of the hearing aid and EEG setup carried in a measurement. A strap connected to the back of the device is used to carry the Portable Hearing Laboratory (PHL) around the neck. Acoustic stimuli are presented *via* the hearing aids connected to the PHL. The around-the-ear EEG sensor cEEGrid is used to measure the neural activity. The cEEGrid is connected to the mobile wireless Smarting EEG amplifier. Both audio and EEG data streams are captured on the PHL for further processing and recording. Figure adapted from Dasenbrock et al. (2021).

This section describes the technical aspects of the setup. For this purpose, the two subsystems, i.e., the PHL and the EEG system, are discussed in more detail. In addition, the LSL framework is discussed in depth, followed by a general system description. Figure 1 outlines how the setup is carried in a measurement.

#### 2.1.1. Portable hearing laboratory

**Figure 2:**
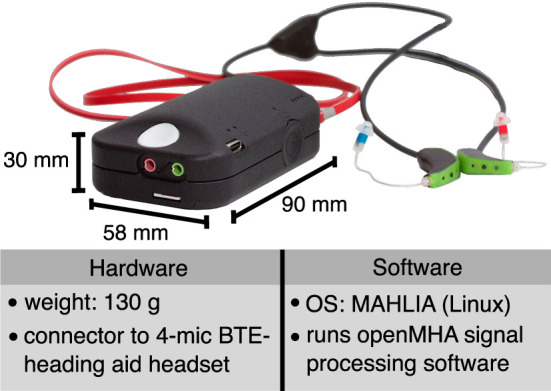
Photo of Portable Hearing Laboratory (PHL) and table with selection of hardware and software features. The hardware consists of a portable main unit and a binaural 4-microphone behind-the-ear (BTE) hearing aid headset. Photo adapted from Kayser et al. (2022).

The PHL is the central component of the setup. It is an integrated hearing aid research platform combining portable hardware and the openMHA. Due to its portability, the device is particularly suitable for field studies to investigate more realistic hearing scenarios outside the laboratory. A personal computer or smartphone controls the PHL through a WiFi connection. It consists of a main unit, including a battery, and supports several ear-level transducers. The setup uses a binaural 4-microphone behind-the-ear (BTE) hearing aid headset developed for the PHL. As visualized in Figure 1, a strap connected to the back of the device is used to carry the PHL around the neck, which weights about 130 g with dimensions of 58 × 90 × 30 mm. The PHL runs a Linux operating system (OS)–MAHALIA (Obbard and James, 2018), optimized for the device's hardware and to run openMHA. The PHL supports sampling rates in the range of 8 and 96 kHz. In this work, a sampling rate of 16 kHz was used. Figure 2 highlights some key features of the PHL's software and hardware. An extended table with detailed technical information on the PHL can be found in the Supplementary material.

openMHA is an open-source software platform for real-time, low-latency hearing aid signal processing (Kayser et al., 2022). The software is implemented in C++ and contains a wide range of audio processing algorithms. Its modular structure allows adding new functionalities in the form of plugins. The functions of a specific openMHA instance (such as algorithms, inputs, outputs, and sampling rate) are defined by the user using the openMHA configuration language consisting of line-based human-readable text commands. The software runs on several computer systems, including lab and portable setups, particularly the PHL.^1^

#### 2.1.2. EEG system

The EEG system used in the presented setup comprises three parts, i.e., (1) electrode setup, (2) Smarting EEG amplifier, and (3) LSL streaming interface. The setup aims to be used mainly with cEEGrid electrodes (Debener et al., 2015; Bleichner and Debener, 2017). The cEEGrid is a disposable electrode grid with printed sensor arrays based on flex-print technology. Ten circular electrodes are arranged in a c-shape to fit around the ear and are attached using adhesive tape. The electrode setup is connected to a small, wireless 24 channel SMARTING EEG amplifier (mBrainTrain, Belgrade, Serbia), placed at the back of the head. The amplifier receives the EEG signal, amplifies it, and streams a Bluetooth signal which is received by the Smarting Android application on the smartphone. The data transfer and synchronization between the Smarting amplifier and the smartphone is handled by a proprietary protocol unknown to the authors. The smartphone and its Smarting app act as the LSL streaming interface. It streams the signal over the network using the LSL framework. In this work, the SMARTING EEG amplifier (Serial number: 010016) is used in combination with a Sony Xperia Z1 smartphone (model: C6903; OS: Android 5.1.1) using a pre-installed Smarting Android application (Version: 1.6.0). A sampling rate of 250 Hz was used. More information on the EEG system can be found in the Supplementary material.

#### 2.1.3. Lab streaming layer

When dealing with multiple components within one setup, each device typically relies on its built-in high-resolution clock. Even if the time difference Δ*t*_clocks_ between the clocks is known at some time, Δ*t*_clocks_ usually changes over time due to clock drift caused by clocks counting at slightly different rates. One possible way of synchronizing data streams of multiple sources is to use hardware synchronization, usually achieved using TTL (transistor-transistor logic) signals, e.g., by regularly sending out synchronization pulses to the attached devices (Reis et al., 2014). However, this usually requires wiring up the different devices. Repeated synchronization of data streams can also be achieved without the need for wires and connectors by using (wireless) networks and a software agent running on each device. For this purpose, the LSL was used. LSL is an open framework that consists of a core library, interfaces for many common programming languages, and several tools (Kothe et al., 2014; Blum et al., 2021). It can be used to measure time differences between connected devices in a network-based setup. Thus, recording setups including LSL can consist of several pieces of hardware and software. LSL tools include a recording program, i.e., the LabRecorder, file importers, and apps to support many EEG systems on the market. The website offers extensive documentation on LSL's functionalities and tools and provides a list of the many different pieces of hardware which have adopted the LSL standard.^2^ The built-in time synchronization capability of LSL is designed for sub-millisecond accuracy on a network of computers connected *via* WiFi.

In LSL terminology, the combination of the raw data from a device and its metadata, such as channel count or sampling rate, is referred to as a stream. LSL streams can have a regular sampling rate, such as continuous EEG, or an irregular sampling rate, such as event markers, e.g., when marking an acoustic event, such as the onset of a sound stimulus. The time synchronization of LSL relies on two pieces of data being collected in addition to the actual sample data: (1) timestamp, (2) clock correction offset. For each LSL sample, a timestamp is read from a local high-resolution clock of the device. The clock correction offset is a measurement of the momentary offset between the two involved clocks and is computed at periodic intervals, by default, every 5 s. LSL is not limited to the use of only two devices. If multiple devices are present in the setup, the clock correction offset between the receiver and every sender is measured.

LSL uses a protocol similar to the Network Time Protocol to measure the clock correction offset. The simplest way to map the time series data from different devices into a common timeline is to add the most recent clock correction offset value to each remotely collected timestamp. Other more sophisticated methods exist that attempt to smooth the clock correction values, such as an outlier-resistant (robust) linear fit through a history of clock correction offsets to reduce the effects of jitter in the clock correction offset measurement. Further, after applying the clock correction offsets, there is a second source of jitter, i.e., jitter in the time stamps. This jitter is not due to synchronization but because time-sampling is usually not done at regular intervals but on a slightly stochastic schedule (determined by the hardware, driver, and operating system). If the LSL stream has a regular sampling rate, this jitter can also be reduced by applying smoothing algorithms. In this work, however, no smoothing of the clock correction offsets and time stamps was applied.

In the setup presented in this work, the LSL framework was used for the synchronization of EEG data and audio event markers. A network link is established by the smartphone's connection (which runs the Smarting app) to the WiFi hotspot provided by the PHL. The possibility of using LSL in the presented setup is provided on the one hand by the Smarting app's functionality to generate LSL streams of the EEG data, and on the other hand by openMHA's interface to LSL. Generally, to use LSL, all clients in the network need to support LSL. Open source Android application projects for LSL streaming and recording enable the inclusion of additional sensor streams (Blum et al., 2021).

#### 2.1.4. System description

**Figure 3:**
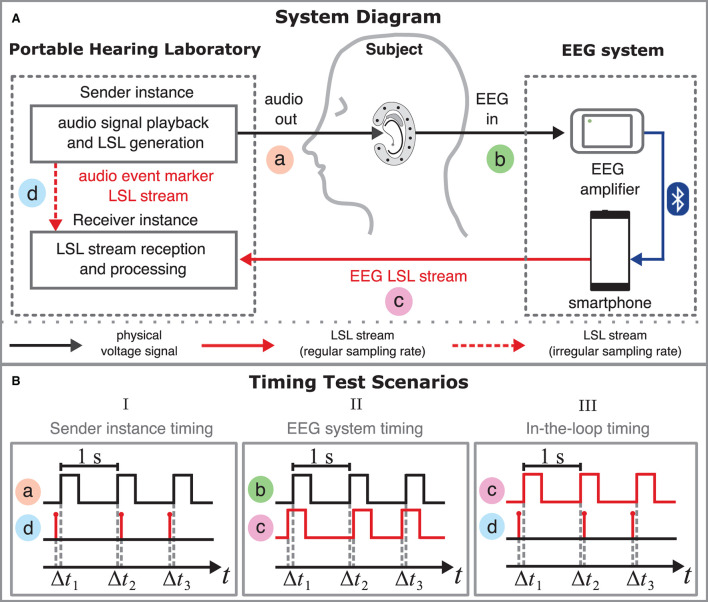
**(A)** System diagram of the measurement setup. The system diagram shows the signal flow and illustrates which components of the setup are responsible for the different signals and data streams and how they are related. Black lines refer to physical voltage signals; red lines refer to LSL streams. The setup combines the Portable Hearing Laboratory (PHL, left) and the EEG system (right). The PHL's function is described in the scheme of a sender-receiver architecture. The sender instance's (top left) role is to present acoustic stimuli *audio out* to the subject *via* the hearing aids. The physical voltage signal is measured at measurement point a. During playback of the stimuli, the sender instance simultaneously creates an *audio event marker LSL stream* that contains event markers indicating specific time points in the audio signal. The *audio event marker LSL stream* is measured at point d. Subsequently, the resulting EEG voltage signal *EEG in* (measurement point b) is amplified using the mobile EEG amplifier. The smartphone receives the EEG data *via* a Bluetooth connection and creates an LSL stream of the EEG data, i.e., *EEG LSL stream* (measurement point c). The receiver instance captures both the *audio event marker LSL stream* and the *EEG LSL stream*. **(B)** Timing diagrams for all three timing test scenarios. Timing diagrams relate two signals or streams in terms of time (x-axis). Square wave signals were used to test the timing in the setup. The time difference between two related time points is defined as trial latencies Δ*t*_*n*_, measured about every second. In timing test Scenario I (left) Δ*t*_*n*_ was computed by comparing the rising edges in the voltage signal *audio out* (a) and the *audio event marker LSL stream* (d). In timing test Scenario II (center) Δ*t*_*n*_ was computed by comparing the rising edges in the voltage signal *EEG in* (b) and the *EEG LSL stream* (c). In timing test Scenario III (right) Δ*t*_*n*_ was computed by comparing the rising edges in the *EEG LSL stream* (c) and the *audio event marker LSL stream* (d).

Figure 3A schematically illustrates which components of the setup are responsible for the different signals and data streams. A total of four measurement points, a-d, were included in the drawing to illustrate between which points the timing was measured. The measurement points will be relevant in Section 2.2, which provides a detailed description of the timing test scenarios.

The setup's data flow is described in the scheme of a sender-receiver architecture running on the PHL (left) and the EEG system (right). The purpose of the sender instance (top left) is to provide acoustic stimuli *audio out* to the subject *via* the hearing aids. The corresponding physical voltage signal is measured at measurement point a. The sender instance simultaneously generates an *audio event marker LSL stream* containing event markers that specify the beginning of a stimulus onset in the audio signal. Point d denotes the position in the data flow at which the *audio event marker LSL stream* is measured.

At measurement point b, the resulting EEG voltage signal (e.g., captured with cEEGrid electrodes) is fed into the EEG amplifier. The smartphone receives the EEG data from the amplifier *via* a Bluetooth connection and creates an LSL stream of the EEG data, referred to as EEG-LSL stream. The *EEG LSL stream* is measured at point c.

The smartphone and the PHL share the same network. This allows the LSL framework to collect all necessary information to map both streams into a common timeline (see Section 2.1.3 for details). Both the *audio event marker LSL stream* and the *EEG LSL stream* are captured by the receiver instance (bottom left). Both sender and receiver instances were implemented as two independent openMHA instances with different processing configurations that run on the PHL.^3^

### 2.2. Timing tests

This section describes the different timing test scenarios performed to investigate the timing properties of the setup outlined in the previous section. It is important to note that the timing tests were performed without humans and an electrode setup, such as the cEEGrid. The signals fed into the EEG system were routed directly into the EEG amplifier. The audio signals from the PHL were also not played directly through the hearing aids but redirected to an audio jack. In each scenario, the timing was evaluated between two of the four measurement points a-d (see Figure 3A). The timing tests of the different scenarios were performed separately, i.e., two different measurement points were measured simultaneously in each scenario.

We conducted tests in three different scenarios. The different scenarios are discussed in more detail in the following sections. An additional test described in Section 2.2.3.1 was performed to compare the recording capabilities of the receiver instance (Figure 3A, bottom left) with an established reference recording software.

**Stimuli**

For all tests, a rectangular pulse was used as a test signal to investigate the time synchronization properties of the setup, as it produces sharp detectable responses. Sixty millisecond pulses were repeated at 1 Hz. All timing test scenarios were performed for two different durations, i.e., 15 min (short) and 3 h (long). For the short timing test 900 and the long timing test, 10,800 trial latencies were determined. A total of 21 timing tests were performed.

#### 2.2.1. Scenario I: Sender instance timing

In timing test Scenario I, the timing accuracy of the sender instance (Figure 3A, top left) was evaluated. The sender instance was programmed to play the test stimulus containing the rectangular pulses. At the same time, it was configured to generate an LSL event marker whenever a rising edge is detected in the signal. More details on the implementation of this mechanism are described in Supplementary Figure 2. In Figure 3B (left), the procedure to test the sender instance's accuracy is sketched in a timing diagram. To specify the timing of the sender instance, the latency Δ*t*_*n*_ was measured. In this scenario, Δ*t*_*n*_ was defined as the time difference between the timestamps of the *audio event marker LSL stream* at measurement point d and the rising edges in the actual playback signal *audio out* at measurement point a.

The LabStreamer (NeuroBehavioral Systems; Albany, CA, USA) was used to measure Δ*t*_*n*_. The LabStreamer is a commercial device that can be considered an oscilloscope optimized for network timing, designed explicitly for analyzing timing precision when dealing with the LSL framework. It provides a sampling rate of 10 kHz, which results in a measuring accuracy of 0.1 ms. It features different input and output channels such as audio, general analog inputs and outputs, and the ability to receive and generate LSL streams. It offers an oscilloscope panel to trigger signals by LSL events and a latency histogram panel. More detailed information on the LabStreamer can be found in the Supplementary material and in the respective documentation.^4^

For the technical implementation, a network connection was established between PHL and LabStreamer to use LSL. The analog signal of the PHL was fed directly into the audio input jack of the LabStreamer. More technical details can be found in Supplementary Section 1.1 of the Supplementary material.

#### 2.2.2. Scenario II: EEG system timing

Timing test Scenario II was done to investigate the timing accuracy of the EEG system (Figure 3A, right) when it converts an analog voltage signal into an LSL stream. Figure 3B (center) visually describes the procedure to test the EEG system's accuracy in a timing diagram. In this scenario, the latency Δ*t*_*n*_ was defined as the time difference between the rising edges in the *EEG LSL stream* at measurement point b and the rising edges contained in the analog *EEG in* test signal fed into the EEG system at measurement point c.

As done in the first scenario, the LabStreamer was used to measure Δ*t*_*n*_. For the technical implementation, a network connection was established between the EEG system and LabStreamer to use LSL, as in timing test Scenario I. The LabStreamer was used to feed the analog test signal into the EEG system and to capture the *EEG LSL stream*. More technical details about this procedure can be found in Supplementary Section 1.2 of the Supplementary material.

#### 2.2.3. Scenario III: In-the-loop timing

In Scenarios I and II, the timing accuracy of the sender instance and EEG system were tested separately. In timing test Scenario III, described in this section, the timing accuracy of the whole setup was measured. For this, the *audio event marker LSL stream* of the sender instance was related to the *EEG LSL stream* of the EEG system. Figure 3B (right) outlines the procedure to test the whole setup's accuracy in a timing diagram. The latency Δ*t*_*n*_ was defined here as the time difference between the timestamps of the sender instance's *audio event marker LSL stream* at measurement point d and the rising edges in the *EEG LSL stream* at measurement point c.

Timing test Scenario III was technically realized by feeding the *audio out* signal of the PHL's sender instance directly into the EEG system as *EEG in* signal (see Figure 3). This approach was adapted from Blum et al. (2017). Further details can be found in Supplementary Section 1.3 of the Supplementary material. In contrast to Scenarios I and II, the LabStreamer was not used here. Both *audio event marker LSL stream* and *EEG LSL stream* were recorded using the receiver instance on the PHL.

The calculation of Δ*t*_*n*_ was done in a post-analysis. The EEG signal was interpolated sample-wise to correspond to the audio signal's sampling rate to determine the rising edge position. This model assumption is justified by the properties of the test signal, which features vertical edges. The EEG signal was epoched from −100 to 150 ms and baseline corrected from −100 to -50 ms with reference to the timestamps in the *audio event marker LSL stream*. As done in Blum et al. (2017), the latency Δ*t*_*n*_ for each rectangular pulse was determined by calculating the time difference between the event marker time and the time when the EEG signal amplitude exceeded the half-maximum of the trial averaged response.

**Metrics**

The lag describes the arithmetic mean within a timing test session, i.e.,


(1) $$\begin{array}{r} \text{lag}=\frac{1}{N}\overset{N}{\underset{n=1}{\sum }}\Delta t_{n}, \end{array}$$

and the jitter the standard deviation of the latency within a timing test session, i.e.,


(2) $$\begin{array}{r} \text{jitter}=\sqrt{\frac{1}{N-1}\overset{N}{\underset{n=1}{\sum }}|\Delta t_{n}-\text{lag}{|}^{2}}. \end{array}$$

An across-session range Δ*R* was calculated to quantify the spread of lag and jitter between timing tests of the same condition. Δ*R* was defined here as the difference between the maximum and the minimum value of lag and jitter across all timing tests of the same condition.

##### 2.2.3.1. Comparison with reference data recording

Two additional 3 h in-the-loop recordings were conducted to compare the data received by the receiver instance with the data received by the standard recording program LabRecorder.^5^ For this, LabRecorder and the receiver instance were used simultaneously on the PHL. The raw LSL data consisting of time series, time stamps, and clock corrections collected from the receiver instance and LabRecorder were compared to check if the receiver instance correctly recorded the data.

## 3. Results

In the following, the results of the previously described timing test scenarios are reported separately. Table 1 gives an overview of the obtained lag, jitter, and the across-session range ΔR of the lag and jitter for each condition.

**Table 1:** Measurement results in terms of lag, jitter, and across-session range ΔR for all three timing test scenarios.

**Duration**	**Meas**.	**Lag**			**Jitter**		
	**Number**	**in ms**			**in ms**		
		**I**	**II**	**III**	**I**	**II**	**III**
15 min	1	32.69	−1.56	29.75	0.07	3.06	1.22
	2	32.76	−53.07	24.92	0.09	3.91	1.5
	3	32.81	−10.5	37.98	0.07	3.02	1.49
	4	32.68	−1.98	29.8	0.09	2.05	1.41
	5	32.67	−21.67	15.36	0.07	1.84	1.24
	ΔR	0.14	51.51	22.62	0.02	2.07	0.28
3 h	1	32.64	−13.2	25.19	0.09	3.82	3.33
	2	32.65	−7.89	24.61	0.09	2.84	2.99
	ΔR	0.01	5.31	0.58	0	0.98	0.34

Δ*R* refers to the difference between the maximum and the minimum value of lag and jitter across all timing tests within one scenario and duration. The columns labeled with Roman numerals belong to the respective timing test Scenarios I–III (see Sections 2.2.1–2.2.3). Five measurements were performed in the 15 min condition **(top)**; two measurements were performed in the 3 h condition **(bottom)**.

### 3.1. Scenario I: Sender instance timing

**Figure 4:**
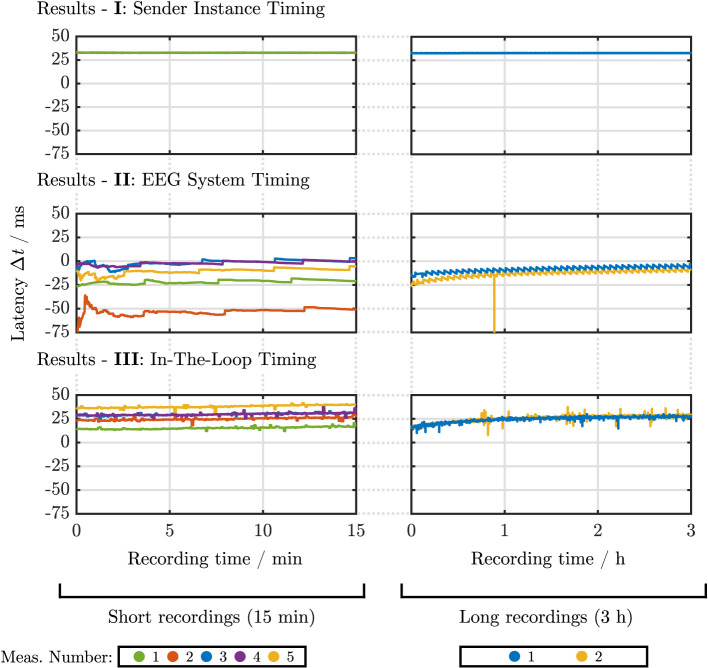
Latency-recording time curves for all timing test scenarios. The different plots show the course of the measured latency (y-axis) in milliseconds over recording time (x-axis) in minutes **(left)** or hours **(right)**. Five measurement runs were performed in the 15 min condition (left column); two measurement runs were performed in the 3 h condition (right column), denoted by different colors. The upper row shows the results for timing test Scenario I: sender instance timing (see Section 2.2.1); the middle row shows the results for timing test Scenario II: EEG system timing (see Section 2.2.2), and the bottom row shows the results for timing test Scenario III: In-the-loop timing (Section 2.2.3).

Figure 4I shows the latency between the LSL timestamps of the *audio event marker LSL stream* and the rising edges in the actual playback signal of the PHL. The Lag was around 32.7 ms for both short and long durations and differed up to 0.14 ms from session to session. Jitters were below 0.1 ms. It can be observed that the latency stayed constant over time for both 15 min and 3 h playback time, i.e., no temporal latency drift was observed.

### 3.2. Scenario II: EEG system timing

Figure 4II presents the latency plotted over recording time between rising edges in the *EEG LSL stream* and the rising edges contained in the analog test signal fed into an EEG system. Contrary to Scenario I the lag did not stay constant, but changed from session to session, indicated by a ΔR of up to 52 ms for the 15 min recording condition. Jitters were, however, relatively constant between sessions, ranging from 1.84 to 3.91 ms and 2.84 to 3.82 ms for the short and long timing test duration. A negative lag was observed in this scenario, which is further discussed in Section 4. Further, a positive, non-monotonic drift can be seen in both cases (short and long timing test duration), i.e., an increasing latency over time. For the 15 min timing test, after substantial fluctuations in the initial 200 s, the latency increases with an approximately linear trend. However, this linear trend no longer persists in the 3 h timing tests, and the latency flattens over time.

### 3.3. Scenario III: In-the-loop timing

Figure 4III shows the latency obtained from the in-the-loop system, i.e., the latency between the timestamps of the sender instance's *audio event marker LSL stream* and the rising edges in the *EEG LSL stream*. Similar to Scenario II lags differed from measurement to measurement with a ΔR of up to 22 ms. Jitters stayed relatively constant within each recording condition, ranging from 1.24 to 1.5 ms and from 2.99 to 3.33 ms for the 15 min and 3 h timing tests, respectively. The latency-recording time plots feature a similar trend as observed in Scenario II, as the recordings show a linear latency drift for the 15 min timing tests, which flattens out over time, visible in the 3 h timing tests.

#### 3.3.1. Comparison with reference data recording

**Figure 5:**
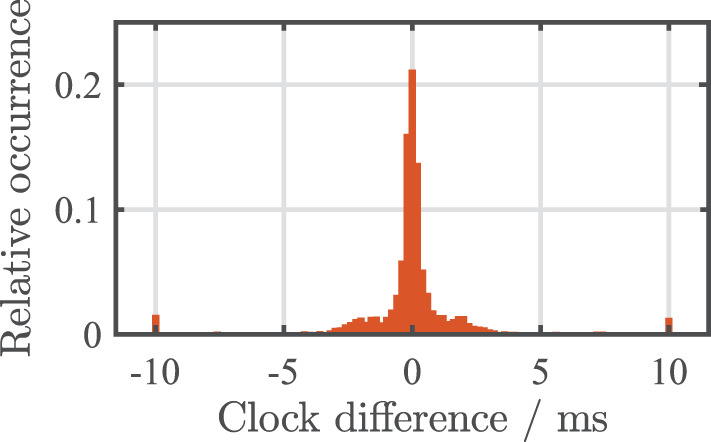
Histogram of one of the two 3 h recordings showing the differences between clock correction offset values obtained using openMHA's receiver instance and LabRecorder. Outlier values outside of ± 10 ms were included in the outer 10 ms bins.

The comparison of receiver instance and LabRecorder data yielded two main results: (1) The raw LSL time series and the locally (in the EEG system and PHL's sender instance) created LSL time stamps were identical for both recording methods. (2) Comparing the LSL clock correction offset values obtained with openMHA and LabRecorder, differences occurred between the two recording methods. However, the long term average amounted to below 0.1 ms for both 3 h measurements, sporadic outliers up to 150 ms occurred for single samples. Figure 5 shows a histogram describing the relative occurrences of clock differences. For illustration purposes outlier values outside of ± 10 ms were included in the outer 10 ms bins.

The slight differences in the obtained clock correction values are negligible on average and can be explained by different points in time at which openMHA and LabRecorder request the clock correction values from the LSL framework. More prominent outliers of the clock correction can usually be eliminated by an outlier resilient fit method of the most recent clock corrections^6^ instead of adding the most recent clock offset value to the time stamp as done in this study for reasons of simplicity.

## 4. Discussion

In this section we compare and discuss the results of the different timing test scenarios. As mentioned in Section 2.1.3 on the LSL framework, no smoothing of clock corrections and time stamps was applied. Hence, the results discussed here can be considered rather conservative and could still be positively influenced by applying jitter-reducing methods.

### 4.1. Scenario I: Sender instance timing

Timing test Scenario I showed a jitter close to the time resolution specified for the LabStreamer of 0.1 ms.^7^ The results show that the PHL can accurately synchronize its audio playback with the information sent over the network using LSL. It should be noted that the lag of the PHL sender instance depends on the sampling rate-fragment size combination. A larger block size with the same sampling rate leads to a higher lag, while the jitter is expected to remain unchanged.

### 4.2. Scenarios II and III: EEG system timing vs. in-the-loop timing

In Scenarios II and III, a jitter of around 3 ms was measured. The increase in jitter compared to Scenario I can most likely be attributed to the EEG system, as it was not used in the first scenario. Variations of the lag from measurement to measurement occurred in both Scenarios II and III involving the EEG system. Even if not further investigated here, these findings align with previous findings when using the Smarting EEG system, which indicates that timing can vary with device, session, and software versions (Debener et al., 2015; Blum et al., 2017). Similar EEG hardware was used in Blum et al. (2017) and Hölle et al. (2022). These studies do not report any drift behavior. One potential reason for this is that in these studies, the *EEG LSL stream* was recorded on the same smartphone, which created the *EEG LSL stream*. In this study, however, the *EEG LSL stream* was recorded by an external device, i.e., the PHL. If the *EEG LSL stream* is recorded on the smartphone, no additional clock correction between PHL and EEG system is required when aligning audio and EEG.

We observed negative latencies in Scenario II. The rising edges in the *EEG LSL stream* were detected before the rising edges contained in the analog test signal. One possible explanation for this behavior is that LSL features the possibility to shift the locally created LSL time stamps by an arbitrary amount, e.g., to compensate for a known latency. If one assumes that the timestamps are generated on the smartphone, a possible explanation for the negative lags is an overcorrection of the timestamps to compensate for the latency caused by the Bluetooth connection. However, the proprietary protocol used for synchronization between Smarting amplifier and smartphone is not accessible, such that the procedure used to convert the data sent *via* Bluetooth into an LSL stream remains unknown. Hence, a more detailed investigation could not be carried out.

Scenarios II and III show very similar latency behavior over time. The two scenarios differ in the fact that Scenario II uses the reference audio signal from the LabStreamer, and Scenario III uses the reference audio signal from the PHL. In both cases, this audio signal is fed into the EEG system. The similar latency-time trend, i.e., a positive, non-monotonic drift, between Scenario II and III indicates that the EEG is causing this latency drift behavior.

The jitter obtained in Scenario II is higher than in Scenario III, presumably attributed to the higher latency fluctuations in the first approx. 200 s of the measurements. As these fluctuations did not occur in Scenarios I and III, they could be traced back to the LabStreamer's function to receive the LSL stream from the EEG system, which may differ from the PHL. One possibility might be that the LabStreamer could have applied online smoothing methods to the clock correction offset values and time stamps received from the EEG system. To record the latencies as purely as possible, the LabStreamers's settings option “Linearize Timestamps” was disabled. This option can be used to reduce the timestamps' jitter by assuming a regular sampling rate of incoming LSL streams (see Section 2.1.3). However, it is unclear if the LabStreamer still applies online smoothing methods to the clock correction offset values. Thus, the initial fluctuations of the latency could be a result of applying online smoothing using a very limited history of clock correction offset values. The exact cause for the fluctuations remains unclear; a more detailed investigation would be interesting but lies outside the scope of the current study.

### 4.3. Comparison with reference data recording

The comparison of the data acquired by the openMHA software with the LabRecorder has shown that data acquisition can accurately be made *via* both ways, i.e., recording with the LabRecorder as well as real-time capturing with the openMHA framework.

### 4.4. Suitability and comparison to state-of-the-art systems

In order to compare the temporal precision of the presented system to existing state-of-the-art technology, it is important to note that there is no standard at which point a mobile EEG system is considered sufficiently precise. Nevertheless, there are some indications that can be used for the evaluation of the system's performance. For instance, Williams et al. (2021) established a jitter threshold, i.e., the point at which jitter made an event-marking method unreliable using an ERP data set recorded using a research-grade EEG system. Their data set contained P100, N100, and P200 peaks. They determined a jitter threshold of 45 ms for the temporally wider P200 peak and a jitter threshold of 16 ms for the temporally sharper N1 peak. They concluded that larger auditory ERP peaks are more robust to jitter, while smaller peaks are more easily mitigated. While the authors point out that these values should not be regarded as absolute thresholds, they can be used as guidelines to narrow down the order of magnitude of temporal requirements for mobile EEG systems. The jitter of the system presented here is well below the jitter thresholds determined in Williams et al. (2021). Further, when comparing the results to existing studies that report timing test data, e.g., Debener et al. (2015); Blum et al. (2017); Mirkovic et al. (2019); Williams et al. (2021); Hölle et al. (2022), the system shows a similar or better accuracy. It can therefore be assumed that the system is suitable to be used for the measurement of temporally sharper peaks, such as the N1. Additionally, in another study, the portable components used for this setup have already been used to record physiological data that enabled the extraction of ERPs, including N100 and P300 peaks, using an Oddball paradigm (Dasenbrock et al., 2021).

### 4.5. Limitations and challenges

It was shown that the jitter of the setup is sufficiently small and thus suitable for online applications. However, the across-session variation of the lag could have a negative influence when averaging over sessions and subjects. The respective impact of the across-session variation of the lag depends on which EEG feature is examined. Temporally wide features that can be spread over several 100 ms, such as the P300 amplitude, can still be decoded between sessions. This can be done using decoders based on 50 ms or 100 ms time bins (Debener et al., 2015; Dasenbrock et al., 2021). Nevertheless, an EEG system with higher lag predictability would facilitate its usability for future applications of the setup.

This study did not examine the extent to which the computational load of PHL might have an impact on the setup's timing accuracy. Factors such as sophisticated signal processing and the addition of more sensors could increase the PHL's CPU or network load. Thus, future studies using the PHL should always investigate the timing for their particular setup. Quick timing tests may be derived from the methods introduced here.

Due to the necessity of a PHL device, the presented setup may be harder to reproduce when compared to fully smartphone-based approaches. However, considering the PHL's specialized hearing aid hardware and openMHA signal processing software, less effort will be required to implement new EEG-based hearing aid algorithms. Regarding their form factor, both PHL and cEEGrid are not entirely suitable for everyday use. In a research context, however, the presented setup provides a useful platform to explore the potential of neuro-steered hearing devices.

### 4.6. Relevance for future applications

Studies such as Zink et al. (2017) or Aroudi et al. (2021) require synchronizing the incoming EEG stream with the audio stream online, as they are received over LSL, e.g., to compare the estimated envelope calculated from the EEG with the envelope of the audio. This requires on-the-fly time synchronization of incoming LSL streams, which is possible with the presented setup, as all information for synchronization is available in the block-based real-time processing in the openMHA software. Further, other approaches exist that incorporate information other than microphone signals into hearing aid processing, based on, e.g., electrooculography (EOG) and head movement sensors to estimate the user's gaze direction (Favre-Felix et al., 2018; Grimm et al., 2018). Since the presented setup uses the well-established LSL framework, other sensors could be integrated in the same manner as employed in this study. This would also allow the setup to be extended to include other sensors besides EEG to expand the setup into a multi-modal research platform (Blum et al., 2021).

## 5. Summary and conclusions

This study performed a comprehensive timing analysis of the research platform introduced in Dasenbrock et al. (2021), focusing on the alignment of audio and EEG data. The setup combines the mobile Smarting EEG system with a portable research hearing device, the Portable Hearing Laboratory (PHL). To perform the timing analysis, we further developed the setup to enable sending single software event markers during the onset of an acoustic stimulus. The temporal precision of the PHL when presenting acoustic stimuli and the EEG system when providing synchronized EEG data was measured using a reference device, i.e., the LabStreamer. Further, after the accuracy of the PHL's stimulus presentation and the EEG system were determined separately, the entire system was examined “in-the-loop” to quantify how the setup's timing accuracy would be in an actual EEG measurement. The data received and recorded with the presented setup were compared to the widely used standard recording program LabRecorder. All timing tests were performed for a short (15 min) and long (3 h) measurement duration. Based on the data collected in this study, we concluded the following:

- The PHL can time-accurately present acoustic stimuli and generate LSL streams over multiple hours of playback.
- The timing accuracy of the EEG system on its own can have a major influence on the overall system's timing. Checking the timing behavior of the EEG system by comparing its LSL stream against a trusted reference is a crucial step when integrating such a system. While the EEG system used in this work is sufficiently accurate within a measurement session, there are noticeable lag variations across sessions. The current setup should be enhanced with an EEG system featuring a higher temporal across-session lag stability to improve its practicability.
- The PHL is capable of presenting acoustic stimuli while simultaneously capturing the audio and EEG LSL streams with sufficient temporal accuracy over multiple hours of playback and recording in an in-the-loop system. The temporal accuracy is sufficient to extract event-related potentials from the EEG.
- Featuring a high temporal precision and real-time signal processing capabilities, the presented setup is suitable as a platform to investigate closed-loop EEG & audio applications for future hearing aids.

## Data availability statement

The data and analysis scripts are publicly available. The raw dataset can be found at https://zenodo.org/record/6857372#.YtWUwi8Rr0o. The analysis scripts as well as the openMHA configurations used in timing test Scenario III are available at https://github.com/steffendasenbrock/SynchronizationEarEEGAudioStreams.

## Author contributions

SDa conceived the experiments with the help of SB, SDe, and HK. SDa carried out the data collection and analysis and wrote the manuscript with the input of SB, PM, SDe, VH, and HK. SDa, PM, and HK implemented the presented setup. All authors contributed to the article and approved the submitted version.

## Funding

This work was funded by the Deutsche Forschungsgemeinschaft (DFG, German Research Foundation) under Germany's Excellence Strategy – EXC 2177/1 – Project ID 390895286.

## Conflict of interest

The authors declare that the research was conducted in the absence of any commercial or financial relationships that could be construed as a potential conflict of interest.

## Publisher's note

All claims expressed in this article are solely those of the authors and do not necessarily represent those of their affiliated organizations, or those of the publisher, the editors and the reviewers. Any product that may be evaluated in this article, or claim that may be made by its manufacturer, is not guaranteed or endorsed by the publisher.

## Footnotes

^1^In this study, the openMHA 4.17.0 release version was used, which is contained in the corresponding MAHALIA image 4.17.0.; MAHALIA download: http://mahalia.openmha.org (accessed March 18, 2022).

^2^More information on the LabRecorder, file importers, and apps can be found in the LSL documentation https://labstreaminglayer.readthedocs.io/index.html (accessed June 25, 2022).

^3^The openMHA configurations for both sender and receiver instances are published and can be found on Github: https://github.com/steffendasenbrock/SynchronizationEarEEGAudioStreams

^4^Neurobehavioral Systems. LabStreamer https://www.neurobs.com/menu_presentation/menu_hardware/labstreamer (accessed June 24, 2022).

^5^LSL. LabRecorder. https://github.com/labstreaminglayer/App-LabRecorder (accessed February 3, 2022).

^6^As, e.g., done in the standard MATLAB importer for .xdf files load_xdf.m: https://github.com/xdf-modules/xdf-Matlab (accessed January 14, 2022).

^7^Neurobehavioral Systems. LabStreamer https://www.neurobs.com/menu_presentation/menu_hardware/labstreamer (accessed March 3, 2022).
