# Supplementary material for: Synchronization of ear-EEG and audio streams in a portable research hearing device
Source: Front Neurosci. 2022 Sep 1;16:904003. doi: 10.3389/fnins.2022.904003 (PMC9475108; doi:10.3389/fnins.2022.904003)
Supplement: Supplementary file 1 [file Data_Sheet_1.PDF]

Supplementary Material

1 SUPPLEMENTARY FIGURES AND TECHNICAL DETAILS

In the following, the technical details for all three timing test scenarios are presented.

1.1 Scenario I: Sender Instance Timing

Figure S1 (panel I) shows a simplified circuit diagram of how the Portable Hearing Laboratory (PHL) and LabStreamer were connected to test the sender instance timing accuracy (timing test Scenario I). The latency Δt_n between the timestamps of the *audio event marker LSL stream* and the rising edges in the *analog audio signal* played back by the PHL's sender instance was measured.

The signal from the PHL's Mini HDMI connector for the hearing aids was picked up using a custom adapter. The analog signal of the PHL was fed directly into the audio jack of the LabStreamer using an audio cable. The LabStreamer received the LSL event markers provided by the sender instance through a network connection [1]. The mechanism used to play audio signals while generating LSL event markers simultaneously is described in Figure S2. The LabStreamer timestamped the analog signal from the PHL and detected the contained rising edges using its build-in mechanism. The LabStreamer's pre-time and post-time were set to $200\,\mathrm{ms}$, defining the amount of time before and after the LSL event marker timestamp, which is searched for a threshold crossing to detect a rising edge. The threshold, determining at which level a rising edge event is detected, was set to half of the peak value of the audio signal input from the PHL. The LabStreamer determined the Δt_n by calculating the time difference between the timestamps of the *audio event marker LSL stream* and the rising edges in the *analog audio signal* played back by the PHL's sender instance. For setting pre-time, post-time, the threshold, and for downloading the latency data, the LabStreamer's web interface was used.

1.2 Scenario II: EEG System Timing

Figure S1 (panel II) shows a simplified circuit diagram of how the EEG system and LabStreamer were connected to test the EEG system's timing accuracy (timing test Scenario II). The latency Δt_n between the rising edges in the *EEG LSL stream* and the rising edges contained in the *analog audio signal* fed into the EEG system was measured.

The smartphone (connected via Bluetooth to the EEG amplifier) of the EEG system was connected to the PHL via WiFi. A network connection between PHL and LabStreamer was established the same way as done in timing test scenario I. Thus, in this timing test scenario, the PHL served only as a device to connect the EEG system and LabStreamer.

The LabStreamer generated a $5\,\mathrm{V}$ rectangular test signal using its digital output. The LabStreamer's voltage signal was reduced using a voltage divider to prevent any clipping or damage to the EEG amplifier. The

[1] In order the receive LSL event markers from the PHL, a network connection was established between PHL and LabStreamer using a WiFi repeater (Fritz!Repeater 1200, AVM GmbH, Berlin, Germany) that created a wireless bridge to the PHL's WiFi. The repeater itself was connected to the LabStreamer via Ethernet.

resulting *analog audio signal* was fed into the EEG system using a cable connected to a *DJ Test Box* adapter provided by Smarting that directs the signal on a single channel of the EEG amplifier.

Synchronous to the *analog audio signal* the LabStreamer created its own internal *audio event marker LSL stream* for the voltage pulses.

The LabStreamer received the *EEG LSL stream* generated by the smartphone. The LabStreamer determined the latency Δt_n by calculating the time difference between the rising edges in the *EEG LSL stream* to the internal *audio event marker LSL stream* of the LabStreamer. The LabStreamer's pre-time and post-time were the same as in timing test scenario I; the threshold was set to half of the EEG LSL stream's peak value.

The smartphone was restarted between the timing tests. Further, the timing tests were started $15\,\mathrm{min}$ after starting the smartphone app's LSL streaming function to prevent impact on the measured data from a strong latency drift behavior of the Smarting system that we observed in the time after the starting the device.

1.3 Scenario III: In-the-Loop Timing

Figure S1 (panel III) shows a simplified circuit diagram of how the PHL and EEG system was connected to test the whole setup's timing accuracy in the loop (timing test Scenario III). The latency Δt_n between the timestamps of the *audio event marker LSL stream* from the sender instance and the rising edges in the *EEG LSL stream* was measured.

The LabStreamer was not used in this timing test scenario. The same sender instance configuration as in timing test Scenario I was used. The *analog audio signal* was fed into the EEG system similarly to timing test Scenario II.

The receiver instance on the PHL collected both *EEG LSL stream* and *audio event marker LSL stream*. The LSL data was stored on the PHL openMHA's recording mechanism. For post-analysis, the data was transferred to a PC to be processed further using custom Matlab scripts to compute the latency Δt_n.

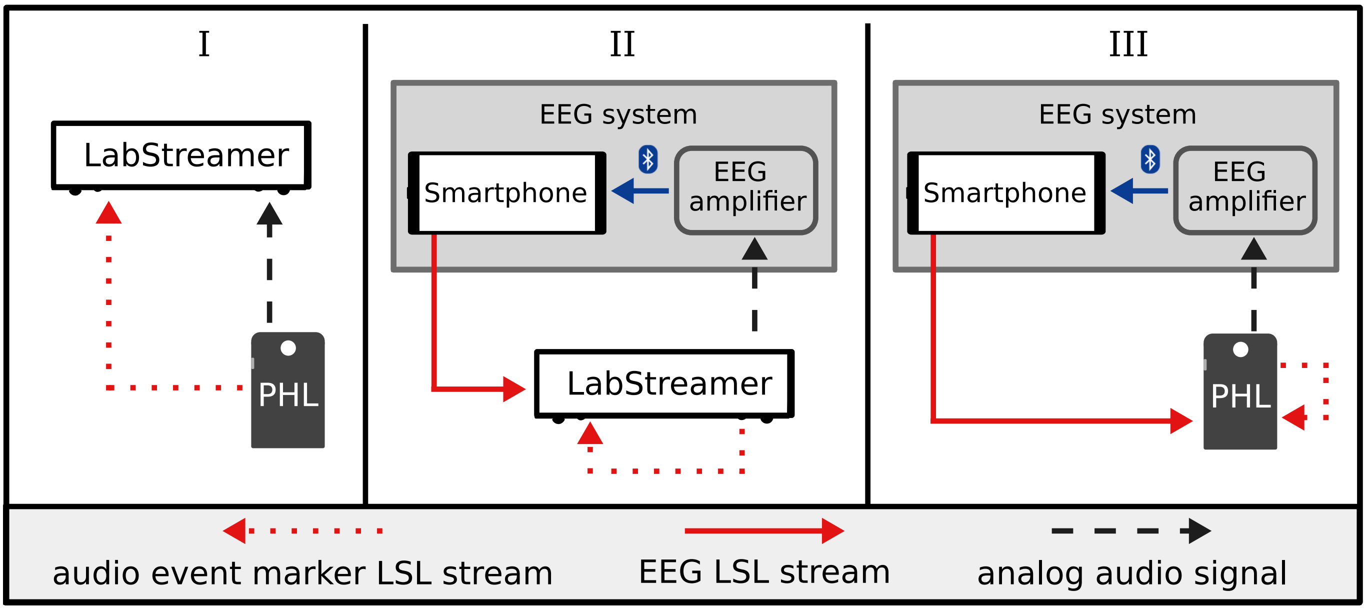

Figure S1. Sketches of all three timing test scenarios for (I) sender instance timing, (II) EEG system timing, (III) in-the-loop timing. **I**: The *analog audio signal* of the PHL was fed directly into the audio input jack of the LabStreamer. The LabStreamer received the *audio event marker LSL stream* provided by the sender instance. **II**: The *analog audio signal* provided by the LabStreamer was routed in the EEG amplifier. The LabStreamer picked up the *EEG LSL stream* provided by the smartphone. **III**: The *analog audio signal* of the PHL was routed in the EEG amplifier. The *EEG LSL stream* provided by the smartphone and the *audio event marker LSL stream* were recorded on the PHL.

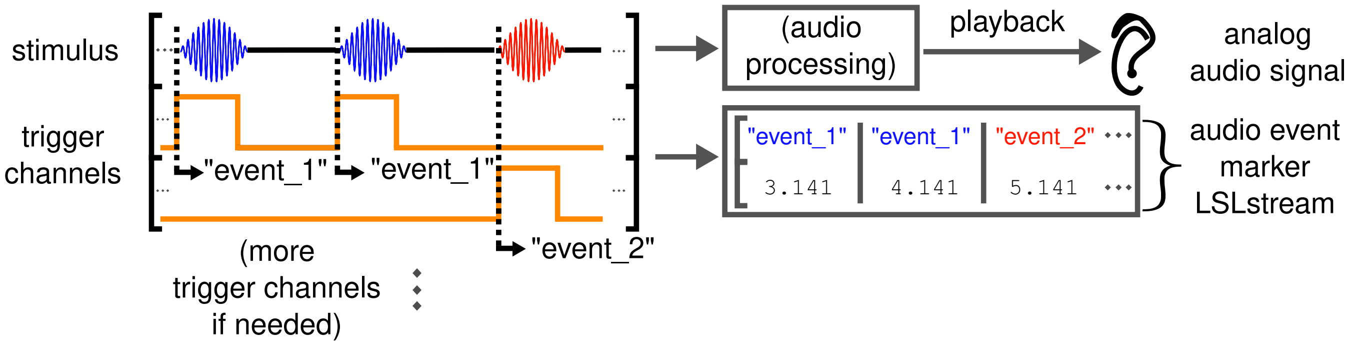

Figure S2. Scheme of a sender instance. The sender instance provides a stimulus to the subject. At the same time, it generates an audio LSL stream containing event markers. Additional audio channels (“trigger channels”), not played to the subject, are used to encode information about, e.g., the stimulus’s onset time and tone type. This information is encoded via rising edges in the trigger channels. Whenever openMHA detects these rising edges, it will send out an LSL event. Note: In the timing test conducted in this study, only one trigger channel was used containing a rectangular signal identical to the stimulus. Thus only one event was present in the timing test setup.

2 DATA SHEET OF USED DEVICES

The following table gives an overview of the technical properties of the devices used in this work. The information was taken from following sources:

- Bat & Cat Soundlabs - PHL user manual
  - `https://batandcat.com/phl-beta---user-manuals.html` [Accessed July 15, 2022]
- Smarting Mobi - user manual
- NeuroBehavioral Systems' website - LabStreamer documentation.
  - `https://www.neurobs.com/menu_presentation/menu_hardware/labstreamer` [Accessed July 15, 2022]

Table S1. Overview of technical properties of devices used in this work. The information was taken from the PHL user manual from Bat & Cat Soundlabs, the Smarting - user manual, and NeuroBehavioral Systems' website of the LabStreamer documentation.

	**Portable Hearing Laboratory**	**EEG System** (amplifier + smartphone)	**LabStreamer**
Manufacturer/ (Version)	Bat & Cat Soundlabs,<br>Palo Alto, California, USA<br>Software: MAHALIA (Debian 10)	mBrainTrain, Belgrade, Serbia<br>Serial number: 010016	NeuroBehavioral Systems,<br>Albany, CA, USA
Sampling rate	8 kHz to 96 kHz, here 16 kHz	250 Hz or 500 Hz, here 250 Hz	10 kHz
Channels	6 input channels total<br>6 output channels total<br>Stereo line-in (3.5 mm)<br>Stereo line-out (3.5 mm)	24 EEG channels<br>One electrode is a reference<br>(unipolar measurement)<br>$\pm$ 100 mV input range	Stereo audio channel input<br>Touch generator<br>Phototransistor<br>4 general analog inputs (-5 to 5 V)<br>8 general digital outputs (0 or 5 V)<br>LSL input stream
Network	Wi-Fi 802.11b/g/n	Wi-Fi 802.11 a/b/g/n/ac (smartphone)	Wired or Wi-Fi 802.11 b/g/n
Bluetooth	Bluetooth 4.1 + BLE	Bluetooth v2.1 + EDR (amplifier)<br>Bluetooth 4.0 + A2DP (Smartphone)	No Bluetooth connectivity
Additional information	- Sitara AM3358 ARM<br>Cortex A8 1000MHz<br>- 512MB DDR3 RAM<br>- 4GB 8 bit eMMC onboard<br>flash storage<br>- External SD card<br>- Built in 2600mAh lithium battery<br>- Dimensions: 58 x 90 x 30 mm<br>- Weight: 130 g<br>- Runs the open Master Hearing Aid<br>(Version: 4.17.0)	Smartphone: Sony Xperia<br>Z1 smartphone<br>(model: C6903; OS: Android 5.1.1)<br>Pre-installed Smarting App<br>(Version: 1.6.0)<br>Electrode connectors: Cristek, IDC<br>Li-Polymer, 560 mAh, 3.7 V<br>Dimensions: 82 x 51 x 12 mm<br>Weight: 60 g	Configuration, control, and display<br>via a web interface:<br>- Oscilloscope Panel<br>- Latency Histogram Panel with statistics<br>- Adjustable channel parameters<br>- Digital output configuration<br>- Latency Chart
